# Development and comparison of predictive models for sexually transmitted diseases—AIDS, gonorrhea, and syphilis in China, 2011–2021

**DOI:** 10.3389/fpubh.2022.966813

**Published:** 2022-08-12

**Authors:** Zhixin Zhu, Xiaoxia Zhu, Yancen Zhan, Lanfang Gu, Liang Chen, Xiuyang Li

**Affiliations:** Department of Epidemiology & Biostatistics, and Center for Clinical Big Data and Statistics, Second Affiliated Hospital, College of Medicine, Zhejiang University, Hangzhou, China

**Keywords:** time series predictive models, sexually transmitted diseases, ARIMA, ERNN, ARIMA-ERNN, LSTM

## Abstract

**Background:**

Accurate incidence prediction of sexually transmitted diseases (STDs) is critical for early prevention and better government strategic planning. In this paper, four different forecasting models were presented to predict the incidence of AIDS, gonorrhea, and syphilis.

**Methods:**

The annual percentage changes in the incidence of AIDS, gonorrhea, and syphilis were estimated by using joinpoint regression. The performance of four methods, namely, the autoregressive integrated moving average (ARIMA) model, Elman neural network (ERNN) model, ARIMA-ERNN hybrid model and long short-term memory (LSTM) model, were assessed and compared. For 1-year prediction, the collected data from 2011 to 2020 were used for modeling to predict the incidence in 2021. For 5-year prediction, the collected data from 2011 to 2016 were used for modeling to predict the incidence from 2017 to 2021. The performance was evaluated based on four indices: mean square error (MSE), mean absolute error (MAE), and mean absolute percentage error (MAPE).

**Results:**

The morbidities of AIDS and syphilis are on the rise, and the morbidity of gonorrhea has declined in recent years. The optimal ARIMA models were determined: ARIMA(2,1,2)(0,1,1)_12_, ARIMA(1,1,2)(0,1,2)_12_, and ARIMA(3,1,2)(1,1,2)_12_ for AIDS, gonorrhea, and syphilis 1-year prediction, respectively; ARIMA (2,1,2)(0,1,1)_12_, ARIMA(1,1,2)(0,1,2)_12_, and ARIMA(2,1,1)(0,1,0)_12_ for AIDS, gonorrhea and syphilis 5-year prediction, respectively. For 1-year prediction, the MAPEs of ARIMA, ERNN, ARIMA-ERNN, and LSTM for AIDS are 23.26, 20.24, 18.34, and 18.63, respectively; For gonorrhea, the MAPEs are 19.44, 18.03, 17.77, and 5.09, respectively; For syphilis, the MAPEs are 9.80, 9.55, 8.67, and 5.79, respectively. For 5-year prediction, the MAPEs of ARIMA, ERNN, ARIMA-ERNN, and LSTM for AIDS are 12.86, 23.54, 14.74, and 25.43, respectively; For gonorrhea, the MAPEs are 17.07, 17.95, 16.46, and 15.13, respectively; For syphilis, the MAPEs are 21.88, 24.00, 20.18 and 11.20, respectively. In general, the performance ranking of the four models from high to low is LSTM, ARIMA-ERNN, ERNN, and ARIMA.

**Conclusion:**

The time series predictive models show their powerful performance in forecasting STDs incidence and can be applied by relevant authorities in the prevention and control of STDs.

## Introduction

In recent years, the attention to sexually transmitted diseases (STDs) has decreased. However, as the main three STDs, AIDS, gonorrhea, and syphilis still cause a severe disease burden globally. AIDS is a highly dangerous infectious disease caused by the human immunodeficiency virus (HIV) attacking the human immune system (1). In 2020, WHO estimated that 1.5 million people acquired HIV and there were an estimated 37.7 million people living with HIV at the end of 2020 (2).

Gonorrhea and syphilis are curable STDs caused by Neisseria gonorrhoeae and treponema pallidum, respectively (3). According to WHO, 82 million new gonorrhea cases and 7.1 million syphilis occurred worldwide in 2020 (4).

In China, the incidence of HIV infection increased annually by 16.3% with 95% confidence interval (CI) of 11.5 to 21.2, and syphilis incidence increased by 16.3% (95% CI: 13.8–18.8), and gonorrhea incidence decreased by 8.5% (95% CI: 11.7–5.1) from 2004 to 2013 (5). AIDS, gonorrhea and syphilis are notifiable diseases according to the Law of the People's Republic of China on Prevention and Control of Infectious Diseases and must be reported to the national infectious disease surveillance system in China once diagnosed (6).

It is of particular importance to actively monitor the morbidity of AIDS, gonorrhea and syphilis, and forecast them accurately. From an ecological research perspective, the most commonly used method for the prediction of trends in infectious disease prevalence is the time-series prediction model. Traditional time series prediction methods realize linear modeling and prediction based on the self-law of time sequence, including the autoregressive integrated moving average model (ARIMA) model (7), gray prediction model (8), exponential smoothing method (9), and Markov model (10). The most representative of these is the ARIMA model, which processes noise and is suitable for short-term prediction of time series, but may result in less than expected prediction accuracy due to its poor non-linear mapping ability. At present, the artificial neural network (ANN) has been applied to epidemic trends prediction of infectious diseases. The widely used feedforward neural network, such as the backpropagation neural network (BPNN) (11, 12), can't well fit the epidemic trend of infectious diseases due to the outbreak, aggregation and variation of infectious diseases.

Elman neural network (ERNN), which has one more acceptor layer than the feed-forward neural network in structure, stores the output state of feedback through a time-delay operator to achieve dynamic memory and internal feedback, which can better fit the epidemic trend of infectious diseases (13). Long short-term memory model (LSTM) is modeled on recurrent neural network (RNN), which avoids the occurrence of RNN's gradient disappearance or gradient explosion. LSTM network is suitable for classifying, processing and predicting time series data. The low requirement for time interval length is an advantage of LSTM over other neural networks (14).

The occurrence of most infectious diseases has a periodic nature. Traditional time series prediction models take the characteristics of periodicity into account, but modeling and prediction are mainly achieved by extracting linear information, and the accuracy needs to be improved. Although performing well in non-linear mapping (15), ANN could not accurately reflect the period of infectious diseases as well as the seasonal variation rules. Both classes of time series prediction models suffer from the issue on incomplete information extraction. Some studies have proposed to combine the above two classes of models to construct hybrid models, such as ARIMA-BPNN (16), ARIMA-GRNN (17), and ARIMA-NAR (18), to simultaneously analyze the characteristics of periodicity and non-linearity of infectious diseases to improve prediction accuracy.

Few studies have explored predictive models for the incidence of STDs in China. In the present study, ARIMA, ERNN, LSTM and ARIMA-ERNN hybrid models were modeled based on the monthly incidence data of AIDS, gonorrhea and syphilis in China from 2011 to 2021 and the performance of each model was compared, to provide a quantitative theoretical basis for STDs prediction and monitoring efforts, and to improve the efficacy in preventing and controlling STDs.

## Materials and methods

### Materials

The monthly incident cases data of AIDS, gonorrhea and syphilis in mainland China reported by the Chinese Center for Disease Prevention and Control from 2011 to 2021 were collected (https://www.phsciencedata.cn/Share/en/). Total number of population at the beginning of the year and total population size at the end of the year in 2011-2021 were collected from Chinese Statistical Yearbook to calculate the average population per year (http://www.stats.gov.cn/tjsj/ndsj/).

### Data analysis

For 1-year prediction, data on AIDS, gonorrhea and syphilis from January 2011 to December 2020 were used as training sets to model respectively, and data from January to December 2021 were used as prediction set. For 5-year prediction, data from 2011 to 2016 were modeled to forecast the incidence from 2017 to 2021. Trend charts were drawn with Excel 2020 and heatmaps were drawn using R 4.2.0 software. Joinpoint Regression Program 4.9.1 software was applied to estimate the annual percentage change (APC). The establishment of the ARIMA model was performed using Eviews 10 software, and the establishment of the ERNN, ARIMA-ERNN, and LSTM model were conducted using Matlab 2022a software. *P*-value ≤ 0.05 was considered statistically significant in this study.

### Methods

#### Trend analysis

The annual percentage change (APC) was estimated by joinpoint regression which focused on estimating the annual changes in the incidence of AIDS, gonorrhea, and syphilis (19). Trend charts and heatmaps were also used to describe the temporal distribution, peak incidence, and periodic variation in the incidence of AIDS, gonorrhea, and syphilis.

#### ARIMA model

The time series model adopted in this study is seasonal time series model ARIMA (*p, d, q*) (*P, D, Q*)*s*, and can be expressed as (20).


(1) $$\begin{array}{l} \nabla^{d}\nabla_{S}^{D}Y_{t}=\frac{\theta_{q}\left(B\right)\Theta_{Q}\left(B^{S}\right)}{\varphi_{p}\left(B\right)\Phi_{P}\left(B^{S}\right)}\varepsilon_{t} \end{array}$$

(2) $$\begin{array}{l} \varphi_{p}\left(B\right)=1-\varphi_{1}B-\varphi_{2}B^{2}-\ldots \varphi_{p}B^{p} \end{array}$$

(3) $$\begin{array}{l} \theta_{q}\left(B\right)=1-\theta_{1}B-\theta_{2}B^{2}-\cdots \theta_{q}B^{q} \end{array}$$

(4) $$\begin{array}{l} \Phi_{P}\left(B^{s}\right)=1-\Phi_{1}B^{s}-\Phi_{2}B^{2s}-\ldots \Phi_{P}B^{Ps} \end{array}$$

(5) $$\begin{array}{l} \Theta_{Q}\left(B^{s}\right)=1-\Theta_{1}B^{s}-\Theta_{2}B^{2s}-\ldots \Theta_{Q}B^{Qs} \end{array}$$

Where, p and q are the non-seasonal autoregressive and moving average order. P and Q are the seasonal autoregressive and moving average order. d is the order of regular differencing and D is the order of seasonal differencing. s is the length of the seasonal period, defined as 12 in present study (21, 22). *B* denotes the backward shift operator, *Y*_*t*_ represents the morbidity of STDs at time t, and ε_t_ are the estimated residuals. In the formula, φ_*p*_(*B*) is the p order autoregressive coefficient polynomial, θ_*q*_(*B*) is the q order moving average coefficient polynomial, $\Phi_{P}\left(B^{s}\right)$ and $\Theta_{Q}\left(B^{s}\right)$ are the seasonal polynomial functions of order P and Q, respectively.

The modeling procedure of ARIMA (*p, d, q*) (*P, D, Q*)*s* model consists of three iterative steps. Firstly, since the time series data are required to be stationary, the stationarity of the time series should be checked by serial plots or the Augmented Dickey-Fuller (ADF) tests (23). For non-stationary data, the stationarity should first be achieved by transformation such as log transformation, and non-seasonal and seasonal differences. Secondly, the autocorrelation function (ACF) graph and partial autocorrelation (PACF) graph were used to determine the possible values of p, d, P, D, and s (24). Subsequently, some unqualified models were removed according to the parametric and residual tests: the parametric test must be statistical significance (*P* ≤ 0.05) and the residual must prove to be a white noise sequence using the ACF and PACF graph of the residual and the Box-Jenkins *Q*-test. Finally, the model with the lowest Akaike information criterion (AIC) and Schwarz Bayesian information criterion (SBC) values was considered the best model (25).

#### ERNN model

Elman neural network is a kind of typical feedback neural network model that has been widely used. It is generally divided into four layers: input layer, hidden layer, recurrent layer and output layer (26). The topology of the ERNN model could be seen in Supplementary Figure 1. The mathematical expression of its network can be expressed as follows:


(6) $$\begin{array}{l} y\left(k\right)=g\left[w_{3}x\left(k\right)\right] \end{array}$$

(7) $$\begin{array}{l} x\left(k\right)=f\left\{w_{1}x_{c}\left(k\right)+w_{2}\left[u\left(k-1\right)\right]\right\} \end{array}$$

(8) $$\begin{array}{l} x_{c}\left(k\right)=x\left(k-1\right) \end{array}$$

Where, *y* is the output node vector; *x* is the hidden layer node unit vector; *u* is the input vector; *x*_*c*_ is the feedback state vector; The *w*_1_, *w*_2_, and *w*_3_ are the corresponding weights.

Major steps to establish ERNN: (1) The “mapminmax” function in Matlab was used to normalize the raw data. (2) The maximum training iterations number and the minimum validation error were set as 1,000 and 10^−6^, respectively. (3) This study chose the following empirical formula for the problem of choosing the number of neurons in the hidden layer:


(9) $$\begin{array}{l} N_{k}=\sqrt{n+m}+a \end{array}$$

where *m* is the number of neurons in the input layer, *n* is the number of neurons in the output layer, and *a* is a constant between 1 and 10. (4) The number of hidden layer neurons with the smallest mean square error (MSE) was selected to construct the ERNN model. (5) The neural network was trained and used to predict and analyze, and the results were back normalized.

#### ARIMA-ERNN model

The model was modeled in a similar way to ERNN, with the core idea of taking the predicted value of ARIMA (*p, d, q*) (*P, D, Q*)*s* model as the input value for ERNN: (1) The optimal ARIMA (*p, d, q*) (*P, D, Q*)*s* model was modeled based on the raw data. (2) The predicted values of the ARIMA (*p, d, q*) (*P, D, Q*)*s* model and the temporal information corresponding to them were normalized together as input datasets. (3) The true values after normalization were taken as the output dataset. (4) Developing an ERNN model for two-dimensional input, and one-dimensional output. (5) The ERNN worked best by continuously learning and training, when the MSE was the smallest. (6) The predictive values of the combined model were back normalized.

#### LSTM model

The input of LSTM is related not only to the current input, but also to the state of the unit. The state of the unit is an accumulation process. LSTM neural network can effectively avoid the disappearance of gradient or gradient explosion. Compared with other neural networks, LSTM is more suitable for time series data prediction. The LSTM unit includes an input gate, a forget gate, and an output gate (Supplementary Figure 2) (27). The LSTM model can be expressed as (28, 29):


(10) $$\begin{array}{l} f_{t}=\sigma \left[W_{f}\times \left(h_{t-1},x_{t}\right)+b_{f}\right] \end{array}$$

(11) $$\begin{array}{l} i_{t}=\sigma \left[W_{i}\times \left(h_{t-1},x_{t}\right)+b_{i}\right] \end{array}$$

(12) $$\begin{array}{l} {\tilde{C}}_{t}=tanh\left[W_{C}\times \left(h_{t-1},x_{t}\right)+b_{C}\right] \end{array}$$

(13) $$\begin{array}{l} C_{t}=f_{t}\times C_{t-1}+i_{t}\times {\tilde{C}}_{t} \end{array}$$

(14) $$\begin{array}{l} o_{t}=\sigma \left[W_{o}\times \left(h_{t-1},x_{t}\right)+b_{o}\right] \end{array}$$

(15) $$\begin{array}{l} h_{t}=o_{t}\times \tanh\left(C_{t}\right) \end{array}$$

where, *x*_*t*_ and *h*_*t*_ are input and output vectors, respectively, *f*_*t*_ is a forget gate vector, *C*_*t*_ represents the cell state vector and it is the input gate vector. *o*_*t*_ is the output gate vector, and *W* and *b* show the parameter matrices. RMSE was used to evaluate the loss of function.

### Model evaluation indices

The accuracy of the four prediction methods used in this study was determined by the comparison between the original observed data and the predicted data obtained by the four methods. In this study, we mainly applied the mean absolute error (MAE), the root mean square error (RMSE), and the mean absolute percentage error (MAPE) to evaluate the fitting and prediction accuracy of the four models. The relative error (RE) reflecting the predictive accuracy of individual month data was calculated as a reference indicator. The equations are as follows:


(16) $$\begin{array}{l} RE=\frac{\left|{ŷ}_{t}-y_{t}\right|}{y_{t}}\times 100\% \end{array}$$

(17) $$\begin{array}{l} MAE=\frac{\overset{n}{\underset{t=1}{\sum }}|{ŷ}_{t}-y_{t}|}{n} \end{array}$$

(18) $$\begin{array}{l} RMSE=\sqrt{\frac{1}{n}\overset{n}{\underset{t=1}{\sum }}{\left({ŷ}_{t}-y_{t}\right)}^{2}} \end{array}$$

(19) $$\begin{array}{l} MAPE=\overset{n}{\underset{t=1}{\sum }}|\frac{{ŷ}_{t}-y_{t}}{y_{t}}|\times \frac{100\%}{n} \end{array}$$

where, ŷ_*t*_ is estimate, *y*_*t*_ is actual value and *n* is sample size.

## Results

### Trend analysis

Table 1 presents that in general, the incidence rates of AIDS, gonorrhea and syphilis were on the rise from 2011 to 2021. The APC was 4.22% (95% CI: 2.37–6.10%), 2.56% (95% CI: 0.24–4.93%), and 2.58% (95% CI: 1.56–3.61%) for AIDS, gonorrhea, and syphilis, respectively, which indicates the incidence rate of AIDS has increased faster than the other two diseases from 2011 to 2021.

**Table 1:** Trends in the incidence of STDs from 2011 to 2021.

**STDs**	**Trend**	**APC (95%CI)**	***t*-value**	***P*-value**
Total	Increase	2.72 (1.63–3.83)	5.67	<0.001
AIDS	Increase	4.22 (2.37–6.10)	5.21	0.001
gonorrhoeae	Increase	2.56 (0.24–4.93)	2.50	0.034
Syphilis	Increase	2.58 (1.56–3.61)	5.78	<0.001

APC, annual percentage change.

**Figure 1:**
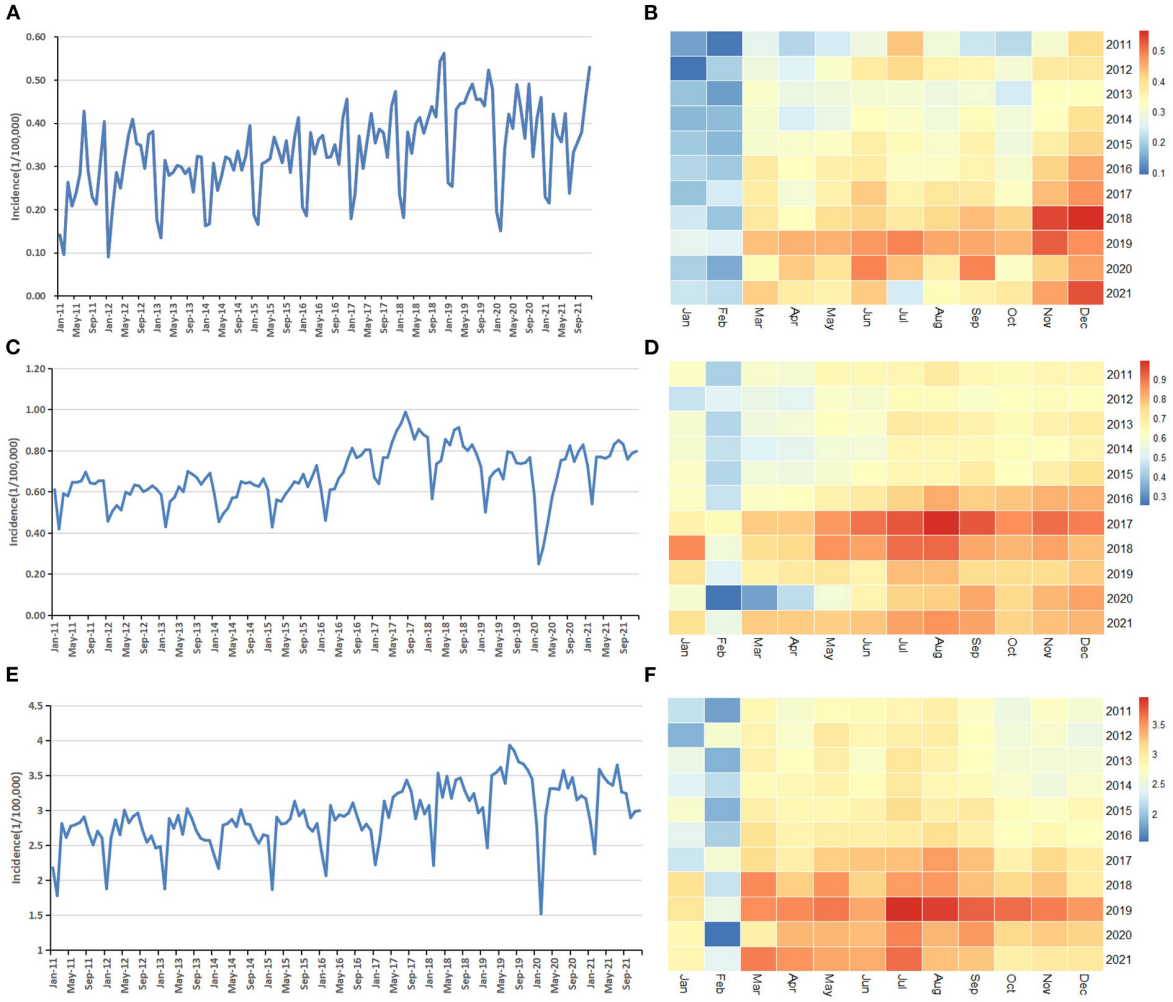
Descriptive analysis of monthly reported incidence of the three STDs: **(A)** Trend chart of AIDS; **(B)** Heat map of AIDS; **(C)** Trend chart of gonorrhea; **(D)** Heat map of gonorrhea. **(E)** Trend chart of syphilis; **(F)** Heat map of syphilis.

Trend charts and heatmaps reflect the overall development trend and periodicity of AIDS, gonorrhea and syphilis incidence, and the incidence rate of gonorrhea has declined in recent years (Figure 1). According to the heat maps, the peak period of AIDS is from November to December, and the peak period of syphilis and gonorrhea is from July to September. The lowest peak periods of the three are all from January to February. All three diseases have seasonality, with a cycle of 12 months.

### ARIMA model

For 1-year prediction, the monthly incidence data of the three STDs from January 2011 to December 2020 in China was used for model fitting. For 5-year prediction, the monthly incidence data of the three STDs from January 2011 to December 2016 in China was used for model fitting. Because of the non-stationarity of the original sequence, a log transformation, non-seasonal (d = 1) and seasonal difference (D = 1) were made to eliminate numerical instabilities. After these steps, the result of the ADF test was statistically significant (), which showed that the time sequence was stationary. The ACF graphs and PACF graphs were used to explore the parameters of the ARIMA (*p, d, q*) (*P, D, Q*)*s* model for 1-year prediction modeling (Supplementary Figures 3A–C) and 5-year prediction modeling (Supplementary Figures 4A–C). The optimal ARIMA models of the three STDs were determined: ARIMA(2,1,2)(0,1,1)_12_, ARIMA(1,1,2)(0,1,2)_12_, and ARIMA(3,1,2)(1,1,2)_12_ for AIDS, gonorrhea and syphilis 1-year prediction, respectively; ARIMA (2,1,2)(0,1,1)_12_, ARIMA(1,1,2)(0,1,2)_12_, and ARIMA(2,1,1)(0,1,0)_12_ for AIDS, gonorrhea and syphilis 5-year prediction, respectively. Parameters in the ARIMA (*p, d, q*) (*P, D, Q*)*s* model(s) were estimated with the conditional least squares method (30). The parameter estimates and test results were showed in Table 2. The ACF graphs and PACF graphs of the residual series (Supplementary Figures 3D–F, 4D–F) suggested the residual series is white noise, so the data was fully modeled.

**Table 2:** Estimate parameters of the ARIMA models for STDs.

**STDs**	**Variable**	**Estimate**	**Standard error**	**t**	**P-value**	**AIC**	**SBC**
**One-year prediction**							
AIDS						−0.533	−0.383
	AR(2)	−0.721	0.159	−4.539	<0.001		
	AR(1)	−0.231	0.106	−2.175	0.032		
	MA(2)	0.763	0.206	3.710	<0.001		
	SMA(1)	−0.677	0.095	−7.111	<0.001		
	Constant	0.003	0.006	−0.449	0.655		
Gonorrhea					−1.376	−1.251	
	AR(1)	−0.421	0.065	−6.506	<0.001		
	MA(2)	−0.516	0.127	−4.050	<0.001		
	SMA(2)	0.273	0.118	2.306	0.023		
	Constant	0.001	0.006	0.233	0.816		
Syphilis						0.207	0.331
	AR(3)	0.279	0.138	2.017	0.046		
	MA(2)	−0.985	0.119	−8.279	<0.001		
	SAR(1)	−0.916	0.070	−13.081	<0.001		
	SMA(2)	0.314	0.128	2.465	0.015		
**Five−year prediction**							
AIDS						−0.311	−0.010
	AR(2)	−0.689	0.191	−3.602	0.001		
	AR(1)	−0.388	0.173	−2.244	0.029		
	MA(1)	−0.646	0.163	−3.959	<0.001		
	SMA(2)	0.639	0.306	2.088	0.042		
	Constant	−0.001	0.009	−0.124	0.902		
Gonorrhea					−2.089	−1.984	
	AR(3)	0.472	0.102	4.620	<0.001		
	AR(2)	0.380	0.092	4.117	<0.001		
Syphilis						−2.052	−1.876
	AR(2)	−0.454	0.140	−3.249	0.002		
	AR(1)	−0.834	0.105	−7.949	<0.001		
	MA(1)	−0.626	0.140	−4.490	<0.001		
	Constant	−0.002	0.002	−0.775	0.442		

SAR, seasonal AR lags; SMA, seasonal MA lags.

### Basic ERNN model and ARIMA-ERNN hybrid model

The training of ANNs for learning seasonality in the data structure does not require any transformation of the original incidence series (31). The period of change in the incidence of the three STDs is 12 months, so the number of neurons in the input and output layers of the ERNN model in this study was 12 and 1, respectively. The number of hidden layer neuronal nodes was calculated according to empirical formula (9) and was determined to range from 4 to 13, which were tested in the network with an increment of 1. The number of hidden layer neurons with minimum MSE was chosen as the optimal number of nodes (Supplementary Table 1). The training target error was 10^−6^ and the learning rate was 10^−3^. Two thousand training sessions were performed. After the training was completed and the network structure was determined, it was used to forecast the incidence iteratively.

For the ARIMA-ERNN model, the predicted values of the ARIMA (*p, d, q*) (*P, D, Q*)*s* model and the temporal information were severed as the input and the actual incidence as the output. The number of neurons in the input layers and in output layers of the ARIMA-ERNN model was 2 and 1, respectively. Different numbers of hidden layer neuronal nodes were also tested (Supplementary Table 1). The training target error, learning rate and training sessions of the ARIMA-ERNN model were identical to those of the ERNN model.

### LSTM model

After many attempts, the optimal LSTM model parameters were finally determined. For AIDS, the epochs, gradient threshold and learning rating were set as 350, 1, and 0.01, respectively. For gonorrhea, the epochs, gradient threshold and learning rating were set as 300, 1, and 0.01, respectively. For syphilis, the epochs, gradient threshold and learning rating were set as 450, 2, and 0.05, respectively, and the optimizer was Adam. The training effect of these models was shown in Supplementary Figure 5. The fact that the curves continue to decline and RMSE drops to 0 before the end of training manifests the neural networks keep learning and achieve the best in the training process.

### Comparisons of the forecasting performance

**Figure 2:**
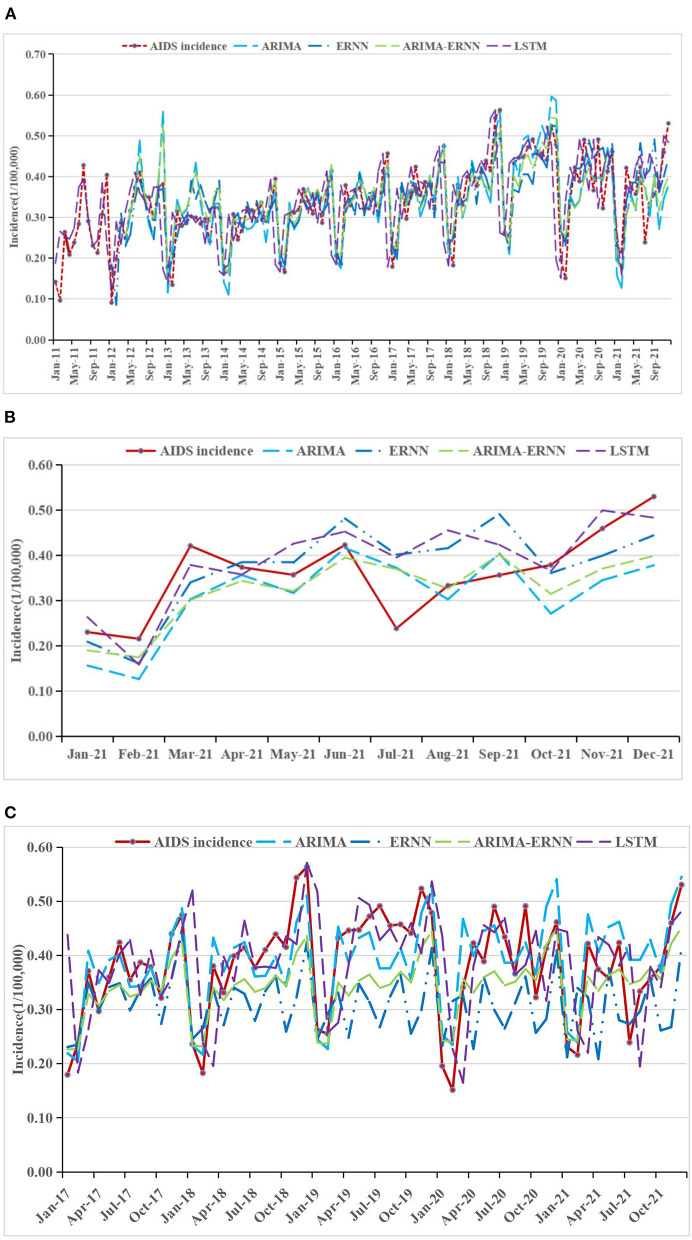
AIDS incidence and fitting values predicted by the four methods **(A)** in 2011–2021, **(B)** in 2021, and **(C)** in 2017–2021.

**Figure 3:**
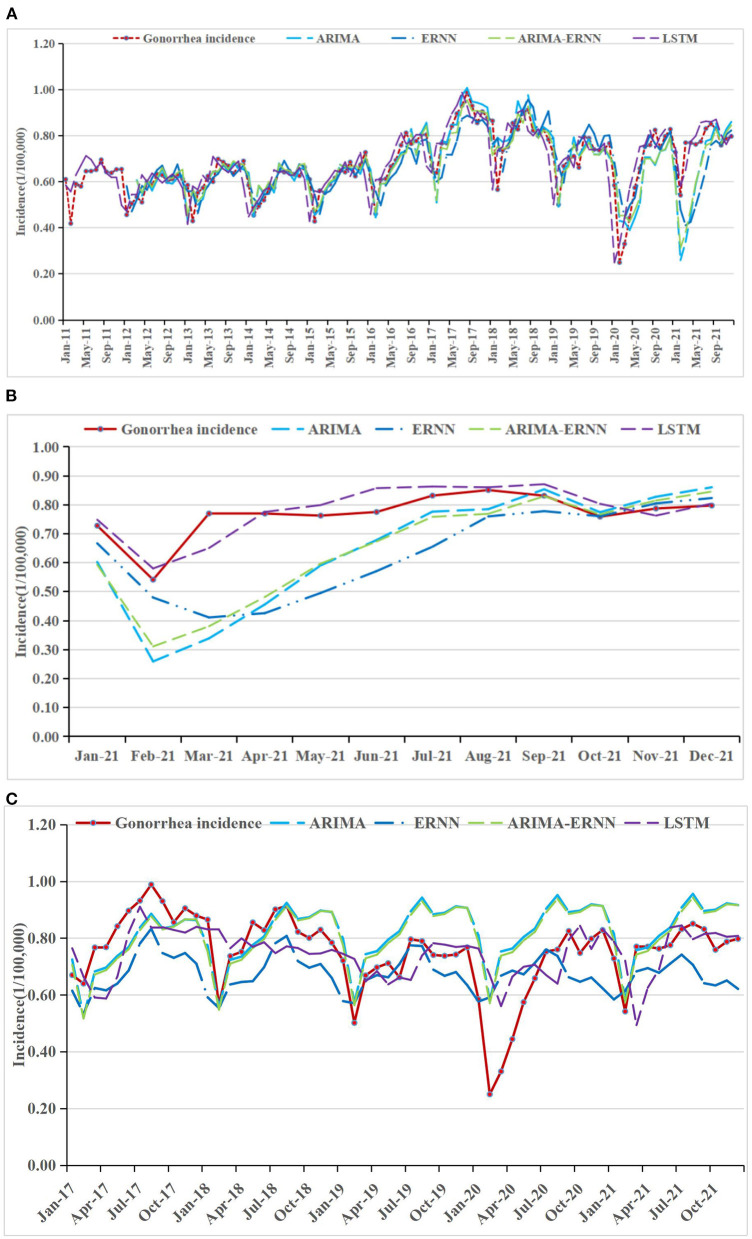
Gonorrhea incidence and fitting values predicted by the four methods **(A)** in 2011–2021, **(B)** in 2021, and **(C)** in 2017–2021.

**Figure 4:**
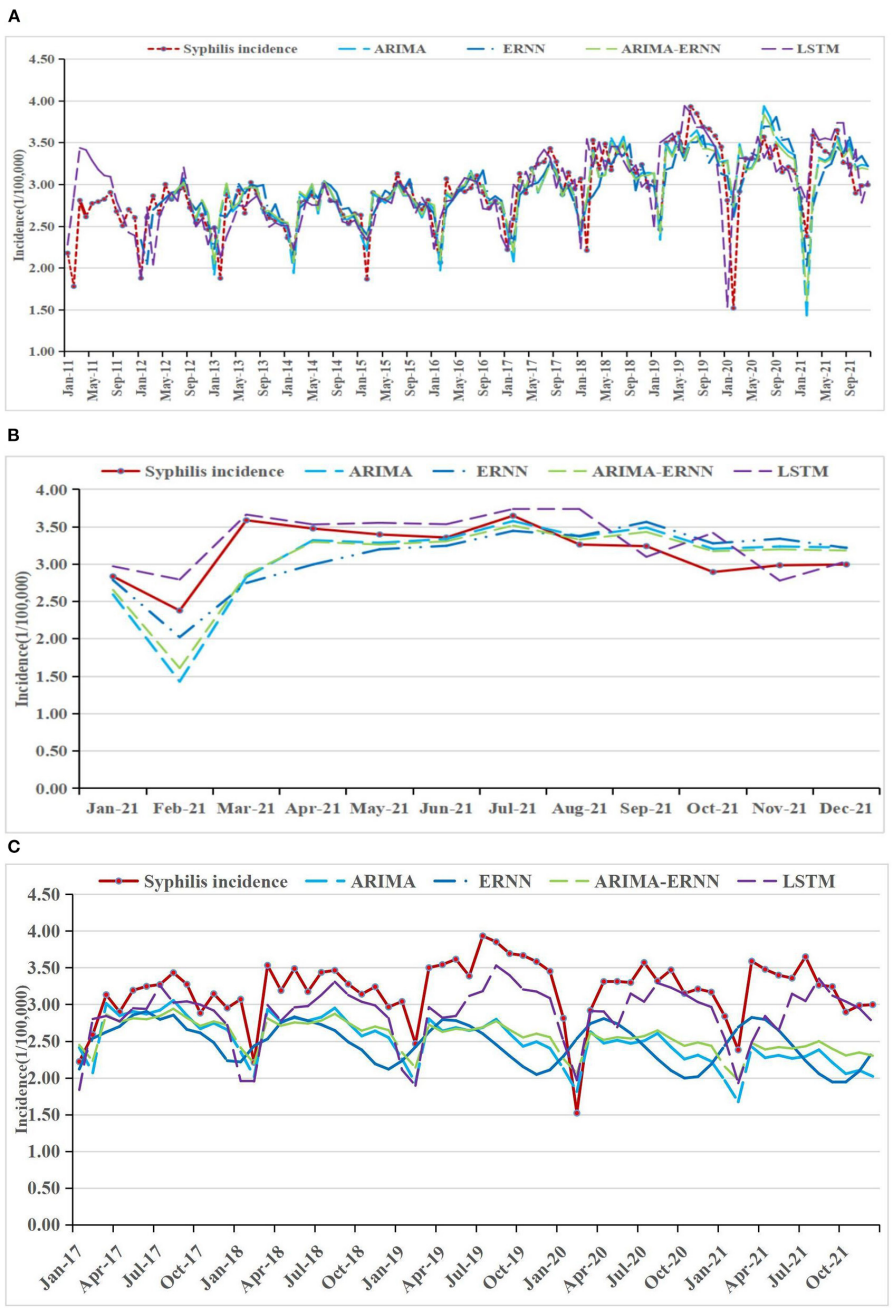
Syphilis incidence and fitting values predicted by the four methods **(A)** in 2011–2021, **(B)** in 2021, and **(C)** in 2017–2021.

The fitting and the forecasting incidences of the four methods were depicted in Figures 2–4. The REs corresponding to each predicted value were listed in Supplementary Table 2. Generally, the fitting values and predicated values obtained by all the four methods reasonably match the actual incidence of the STDs.

Table 3 showed the modeling and prediction performances of the four models.

**Table 3:** Comparison of the performances of the four different models^a^.

**STDs**	**Model**	**Modeling performance**			**Predicting performance**		
		**MAE**	**RMSE**	**MAPE (%)**	**MAE**	**RMSE**	**MAPE (%)**
AIDS	ARIMA	0.04/0.05	0.05/0.03	12.00/11.87	0.08/0.05	0.09/0.05	23.26/12.86
	ERNN	0.04/0.04	0.05/0.05	12.36/14.42	0.07/0.09	0.08/0.11	20.24//23.54
	ARIMA-ERNN	0.03/0.03	0.04/0.04	11.00/9.72	0.06/0.06	0.08/0.07	18.34/14.74
	LSTM	0.07/0.03	0.09/0.04	23.39/11.00	0.06/0.08	0.07/0.11	18.63/25.43
Gonorrhea	ARIMA	0.04/0.04	0.06/0.03	7.25/5.08	0.14/0.13	0.19/0.10	19.44/17.07
	ERNN	0.05/0.05	0.07/0.06	8.50/8.23	0.14/0.12	0.19/0.14	18.03/17.95
	ARIMA-ERNN	0.04/0.03	0.06/0.04	6.75/4.98	0.13/0.10	0.17/0.13	17.77/16.46
	LSTM	0.01/0.33	0.02/0.06	1.38/5.95	0.04/0.09	0.05/0.12	5.09/15.13
Syphilis	ARIMA	0.16/0.18	0.25/0.14	6.15/5.40	0.29/0.78	0.39/0.71	9.80/21.88
	ERNN	0.18/0.26	0.25/0.30	6.775/9.744	0.30/0.78	0.13/0.87	9.55/24.00
	ARIMA-ERNN	0.16/0.12	0.24/0.16	6.079/4.605	0.26/0.66	0.35/0.72	8.67/20.18
	LSTM	0.23/0.21	0.12/0.31	8.571/8.932	0.17/0.35	0.25/0.44	5.79/11.20

^a^One-year forecast performance/5-year forecast performance.

MAPE was used as the primary model performance measure for STDs, because it is a relative index among the three evaluation indices. For 1-year prediction, for AIDS, there is no significant difference between the performances of the models. It can be seen that the MAPE in ARIMA-ERNN model is the lowest among the four models in predicting (18.3%), and the MAE and RMSE are also relatively low; LSTM has a good predicting performance (18.6%) while its modeling performance is relatively poor (23.4%). For gonorrhea, the performance of LSTM is significantly better than other models, with the lowest MAPE, MAE and RMSE for both modeling (1.4%) and predicting data (5.1%); ARIMA-ERNN is the second-best among these models, and ERNN and ARIMA (*p, d, q*) (*P, D, Q*)*s* models performed almost equally. For syphilis, LSTM performs well in predicting (5.8%), and ARIMA-ERNN has better modeling performance (6.1%). Compared with 1-year prediction, the performance of 5-year prediction is slightly worse, mainly in the prediction of syphilis.

In general, these models have good performance both in long-term prediction and short-term prediction. For modeling performance, the MAPEs of these models are close to or less than 10%, indicating that the fitting effect is good and there is no underfitting. For prediction performance, the MAPEs are close to or less than 20%, which is not much different from the MAPEs for modeling performance, indicating that the prediction effect is good and there is no overfitting. In terms of prediction, the MAPEs of LSTM are smaller than those of the other models, and the overall performance of the four models was ranked in descending order as follows: LSTM, ARIMA-ERNN, ERNN, and ARIMA (*p, d, q*) (*P, D, Q*)*s*.

## Discussion

STDs are the most common infectious diseases in the world. Although STDs are largely preventable, they continue to cause serious incidence rate and mortality. STDs surveillance remains a key component of global surveillance and response. Through the analysis of reliable monitoring information, the planning of prevention and treatment strategies can be evaluated in time, so that the project adjustment, advocacy, strategic planning and resource mobilization will be optimized (32).

The factors associated with the prevalence of STDs include engagement in unsafe sexual practices, especially among some special populations, such as men who have sex with men, and female sex workers and their clients (33, 34). STDs are not seasonal infectious diseases, but this study reveals a periodicity pattern for the incidence of the three STDs in China, this phenomena can be related to sexual behaviors in Chinese populations, the impact of seasonal migration in China, and the patients' clinical attendance (35). Previous studies have also shown that rural-to-urban migration, social stigma, and lack of healthcare-seeking behavior expand the spread of HIV/AIDS and syphilis (34, 36–38). Since AIDS, gonorrhea and syphilis share similar risk factors and can be co-transmitted, it is reasonable to analyze their epidemiological characteristics together and take combined interventions to control the prevalence of them (39, 40).

The incidence of AIDS, gonorrhea and syphilis reached their lowest during the January and February of each year but then quickly rose to a relatively high level. This may be due to the unique effects of the annual Chinese New Year which generally falls in late January or early February, during which national and provincial CDCs are not fully functional and most hospital labs run on limited capacity, resulting in the artificial drops in STDs incidence records (22). It was observed that the incidence of the currently studied STDs decreased dramatically in January 2020, when the COVID-19 pandemic just broke out. A previous study found that the sharp decline in STDs incidence was maintained almost 5 months after the lockdown started because of the COVID-19 pandemic (41). But according to the trend charts in this study, the COVID-19 pandemic seemed to have no influence on the general trend and periodicity of AIDS, gonorrhea and syphilis incidence, so it has little impact on our modeling effect.

A perfect surveillance system helps researchers to collect and analyze infectious disease data. With high-quality surveillance data, the epidemic behavior may be accurately detected and forecasted. The data in this study were the latest and collected from the Chinese Center for Disease Prevention and Control which has the most authoritative infectious disease surveillance system in China. So, the data quality and authenticity can be guaranteed.

Several researches had been done to introduce different approaches to forecasting epidemic incidence. The ARIMA (*p, d, q*) (*P, D, Q*)_*s*_ model was used to model and predict the incidence of influenza and mumps in China and performed well (5, 7). The ARIMA (*p, d, q*) (*P, D, Q*)_*s*_ model is popular because of its known statistical properties and the well-known Box–Jenkins methodology in the modeling process, but it can only extract linear relationships within the time series data and may not work well for the occurrence of an infectious disease which can be affected by various factors. The ANN time series models capture the historical information by non-linear functions. ERNN model was reported to have a better performance than BPNN and ARIMA (*p, d, q*) (*P, D, Q*)_*s*_ model in forecasting typhoid fever incidence in China (13). For hybrid models, the hybrid ARIMA-GRNN model showed better hepatitis incidence forecasting in Heng County than the single ARIMA (*p, d, q*) (*P, D, Q*)_*s*_ model and the basic generalized regression neural network (GRNN) model (17). LSTM model has demonstrated better performance than BPNN in forecasting hepatitis incidence in China (42), and better than the recurrent neural network in forecasting COVID-19 in Malaysia, Morocco and Saudi Arabia (43). The different findings of these studies suggest that further studies comparing different kinds of forecasting methods for different kinds of diseases are necessary for the application in predicting epidemic behavior.

The effectiveness of statistical models in forecasting future STDs incidence has been recognized (44). Common prediction models for STDs include ARIMA model, great prediction model, exponential smooth model, BPNN model and GRNN model. Similar to the ARIMA model, the exponential smoothing model is also a linear statistical model. It assumes greater predictive value for recent observations than for earlier ones and gives greater weight to the former (45). Gray prediction model is used to investigate a large amount of unknown information using a small amount of information in a system containing incomplete data, which is widely used due to its virtue of “strong adaptability, simple model, easy parameter changes” (46). Exponential smooth and gray models are generally good for short-term predictions, but they tend to perform poorly in long-term predictions (47). BPNN and GRNN models are belong to artificial neural network, and their advantages are that they have better non-linear mapping ability to obtain good prediction accuracy, but the statistical significance of the models is unclear, and the interpretability of the parameters is inferior to some statistical models such as ARIMA model and exponential smooth model (11). Several studies have developed predictive models for the incidence of STDs are still rare. Wang et.al concluded that the LSTM model was a better predictive model than the ARIMA (*p, d, q*) (*P, D, Q*)_*s*_, GRNN and exponential smoothing model in forecasting the HIV incidence in Guangxi, China (47). Li et.al reported the BPNN model was a more suitable method than the ARIMA (*p, d, q*) (*P, D, Q*)_*s*_ model to monitor and predict the changing trend and morbidity of AIDS in China (48). But the data collected in the above two studies was relatively early. In Mao's research, ARIMA (*p, d, q*) (*P, D, Q*)_*s*_ model had a good precision in predicting syphilis incidence in China, but it only had short-term forecasts for 6 months (49). Though Xu et al. used ARIMA (*p, d, q*) (*P, D, Q*)_*s*_ to model both the incidence and mortality of AIDS, they didn't make a comparison between the effectiveness of different models (22). Ye et al. comprehensively model the incidence of AIDS, gonorrhea, and syphilis in China with a gray model, and made a good prediction, yet they used annually rather than monthly incidence (8).

Compared with previous studies, the current study has several innovations and strengths. First, since the incidence of AIDS, gonorrhea, and syphilis shared similar seasonal patterns, it is feasible to analyze them together, develop different prediction models and compare their performance to obtain more generalizable optimal time series prediction models that can be applied to STDs. Second, the performance of four models with different features, namely, the ARIMA model based on traditional linear statistical methods, the traditional neural network ERNN model, the hybrid model, and the burgeoning deep learning LSTM model, were comprehensively analyzed and compared, which have their own different advantages and are more representative. Finally, both short-term (1-year) and long-term (5-year) forecasts were conducted to comprehensively explore the performance of these models.

Time series prediction models have their particular advantages. First, they are able to make full use of the temporal information of the original dataset to make accurate predictions. Second, the modeling process is not complicated, so the models can be generalized for use. Finally, model parameters can be dynamically optimized by incorporating recently reported data to facilitate timely disease prediction. Due to the advantages and good performance of the models, the time-series models studied in this research can be used to predict peak incidence of AIDS, gonorrhea, and syphilis, so that relevant authorities such as the CDC can prepare for it as early as possible and take countermeasures, which will optimize the prevention and control effects of STDs and resource mobilization.

The limitations of the study should also be acknowledged. First, we only collected national data on AIDS, gonorrhea and syphilis, but did not collect data from different provinces and cities. Therefore, we lacked analysis on this part. However, this study is still informative for the modeling of STDs incidence at the provincial and regional levels, because in general, the temporal regularity of STDs incidence is similar. Second, the findings based on a specific disease may not be repeatable when used in other cases. Third, the epidemic of STDs is influenced by many elements, such as environmental changes, human behaviors and health interventions. The single factor model may be not compatible with complex epidemic problems.

Based on the above limitations, we make the following suggestions for future research. First, some advanced neural network algorithms such as arithmetic optimization algorithm and genetic algorithm can be applied to optimize neural network modeling. Second, it is possible to develop ARIMAX model, panel data prediction model, and multi-input layer neural network on the basis of ARIMA model and traditional neural network model by adding spatial information or other covariates, thus improving the prediction accuracy.

## Conclusion

With good performance, the ARIMA (*p, d, q*) (*P, D, Q*)_*s*_ model, ERNN model, ARIMA-ERNN model, and LSTM model can be applied to forecast the incidence of AIDS, gonorrhea, and syphilis and have the potential to help the department concerned make efficient decisions to significantly promote STDs control and management.

## Data availability statement

The datasets presented in this study can be found in online repositories. The names of the repository/repositories and accession number(s) can be found at: http://www.nhc.gov.cn/jkj/s2907/new_list.shtml?tdsourcetag=s_pcqq_aiomsg.

## Author contributions

XL and ZZ conceived and designed the study. XZ, LG, YZ, and LC extracted the data. ZZ and XZ analyzed the data. ZZ contributed analysis tools and wrote the manuscript. XL revised the manuscript. All authors contributed to the article and approved the submitted version.

## Funding

This work was supported by the Soft Science Key Project of the Science and Technology Department of Zhejiang Province (2019C25009 and 2022C25040), the Key Project of Social Science Planning in Hangzhou City (hzjz20180110), and the Soft Science Key Project of Hangzhou Municipal Science Committee (20160834M03).

## Conflict of interest

The authors declare that the research was conducted in the absence of any commercial or financial relationships that could be construed as a potential conflict of interest.

## Publisher's note

All claims expressed in this article are solely those of the authors and do not necessarily represent those of their affiliated organizations, or those of the publisher, the editors and the reviewers. Any product that may be evaluated in this article, or claim that may be made by its manufacturer, is not guaranteed or endorsed by the publisher.

## Supplementary material

Supplementary Figure 1 Schematic of ERNN.

Supplementary Figure 2 Schematic of LSTM.

Supplementary Figure 3 ACF and PACF graphs of the post differential and residual sequences from 2011 to 2020: **(A)** ACF and PACF graphs of the AIDS data (d = 1 and D = 1), **(B)** ACF and PACF graphs of the gonorrhea data (d = 1 and D = 1), **(C)** ACF and PACF graphs of the syphilis data (d = 1 and D = 1), **(D)** ACF and PACF graphs of the residential sequence from AIDS data, **(E)** ACF and PACF graphs of the residential sequence from gonorrhea data, and **(F)** ACF and PACF graphs of the residential sequence from syphilis data.

Supplementary Figure 4 ACF and PACF graphs of the post differential and residual sequences from 2011 to 2016. **(A)** ACF and PACF graphs of the AIDS data (d = 1 and D = 1). **(B)** ACF and PACF graphs of the gonorrhea data (d = 1 and D = 1). **(C)** ACF and PACF graphs of the syphilis data (d = 1 and D = 1). **(D)** ACF and PACF graphs of the residential sequence from AIDS data. **(E)** ACF and PACF graphs of the residential sequence from gonorrhea data. **(F)** ACF and PACF graphs of the residential sequence from syphilis data.

Supplementary Figure 5 Training charts of LSTM model. **(A)** One-year prediction for AIDS. **(B)** Five-year prediction for AIDS. **(C)** One-year prediction for gonorrhea. **(D)** Five-year prediction for gonorrhea. **(E)** One-year prediction for syphilis. **(F)** Five-year prediction for syphilis.
