# Supplementary figures and images for: Development and comparison of predictive models for sexually transmitted diseases—AIDS, gonorrhea, and syphilis in China, 2011–2021
Source: Front Public Health. 2022 Aug 12;10:966813. doi: 10.3389/fpubh.2022.966813 (PMC9450018; doi:10.3389/fpubh.2022.966813)

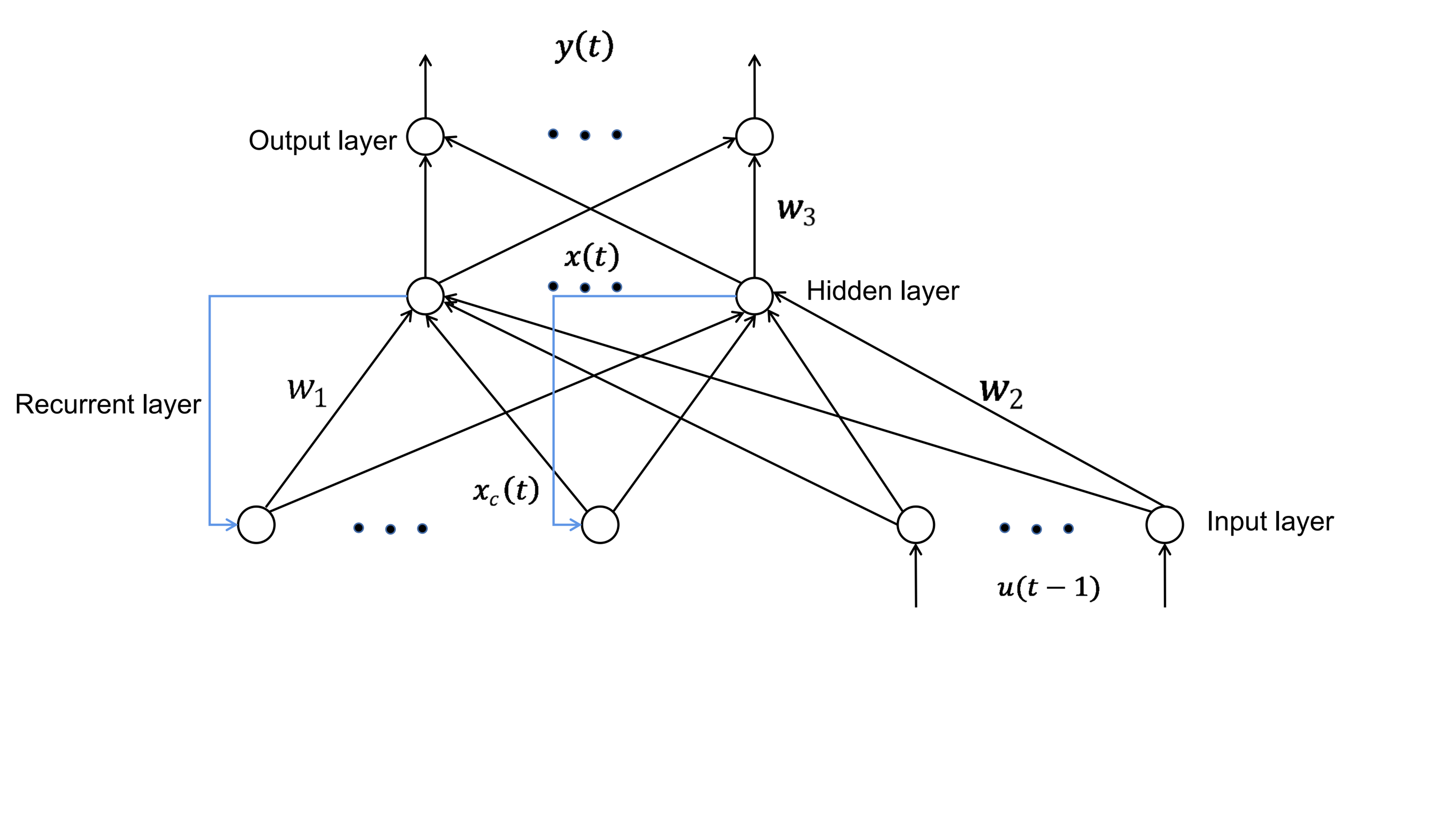

Supplement: Supplementary Figure 1 — Schematic of ERNN. [file Image_1.JPEG]

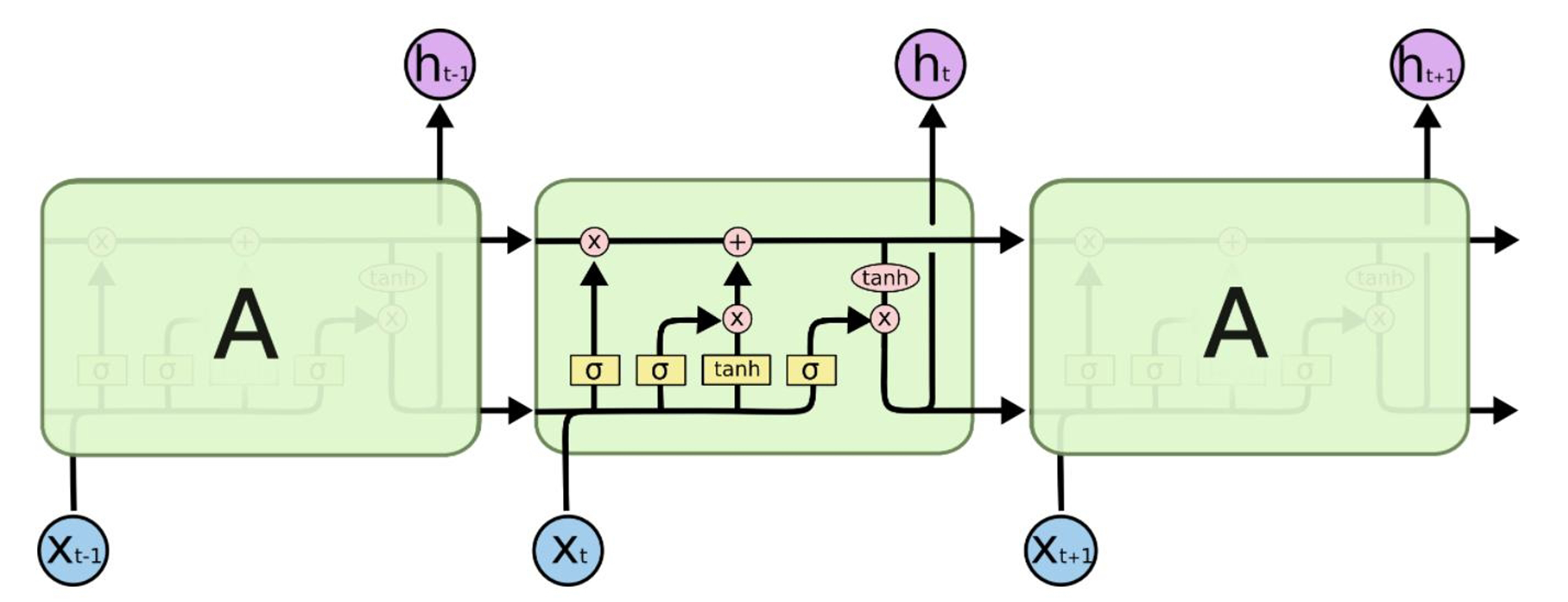

Supplement: Supplementary Figure 2 — Schematic of LSTM. [file Image_2.JPEG]

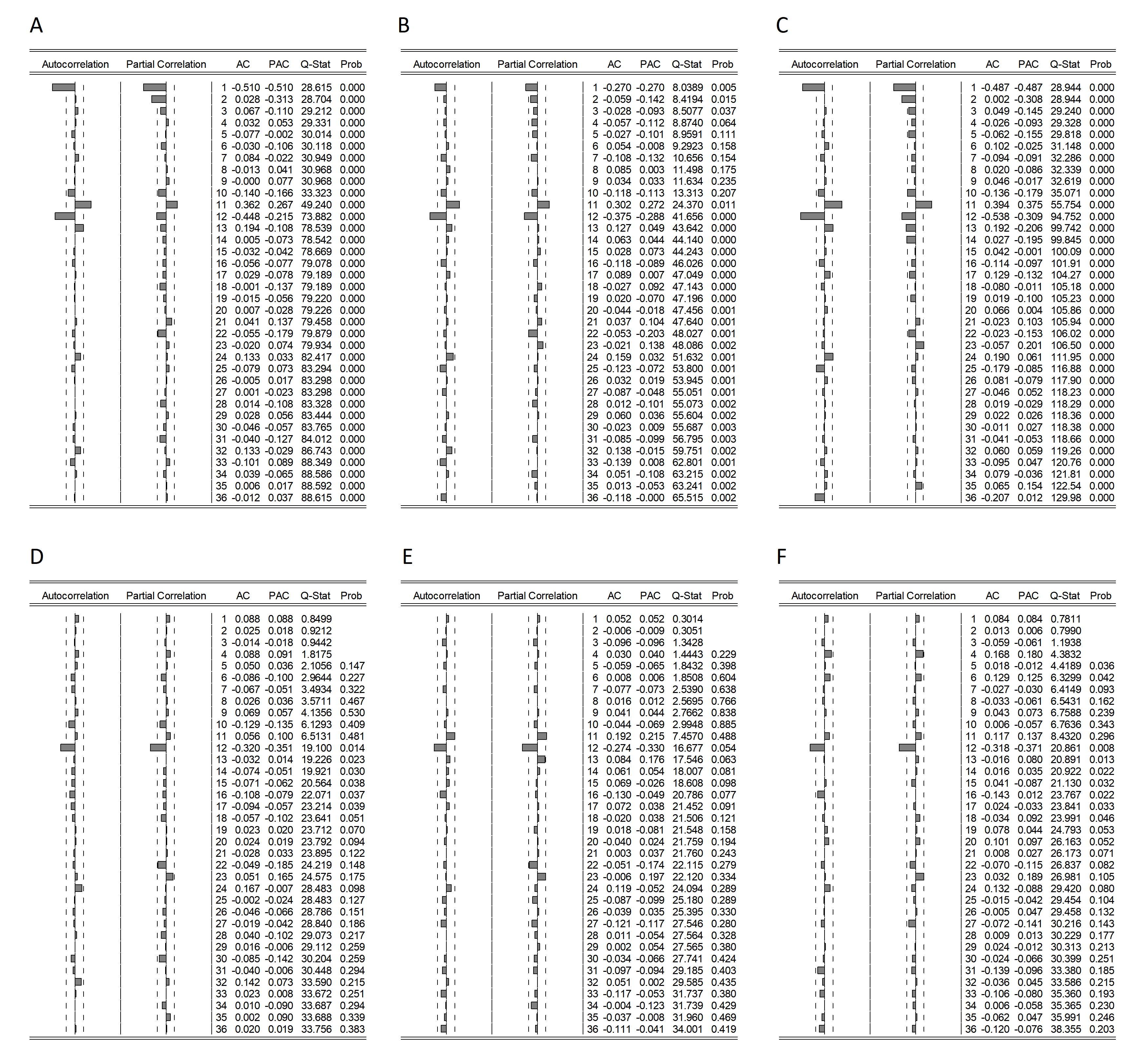

Supplement: Supplementary Figure 3 — ACF and PACF graphs of the post differential and residual sequences from 2011 to 2020: (A) ACF and PACF graphs of the AIDS data (d = 1 and D = 1), (B) ACF and PACF graphs of the gonorrhea data (d = 1 and D = 1), (C) ACF and PACF graphs of the syphilis data (d = 1 and D = 1), (D) ACF and PACF graphs of the residential sequence from AIDS data, (E) ACF and PACF graphs of the residential sequence from gonorrhea data, and (F) ACF and PACF graphs of the residential sequence from syphilis data. [file Image_3.JPEG]

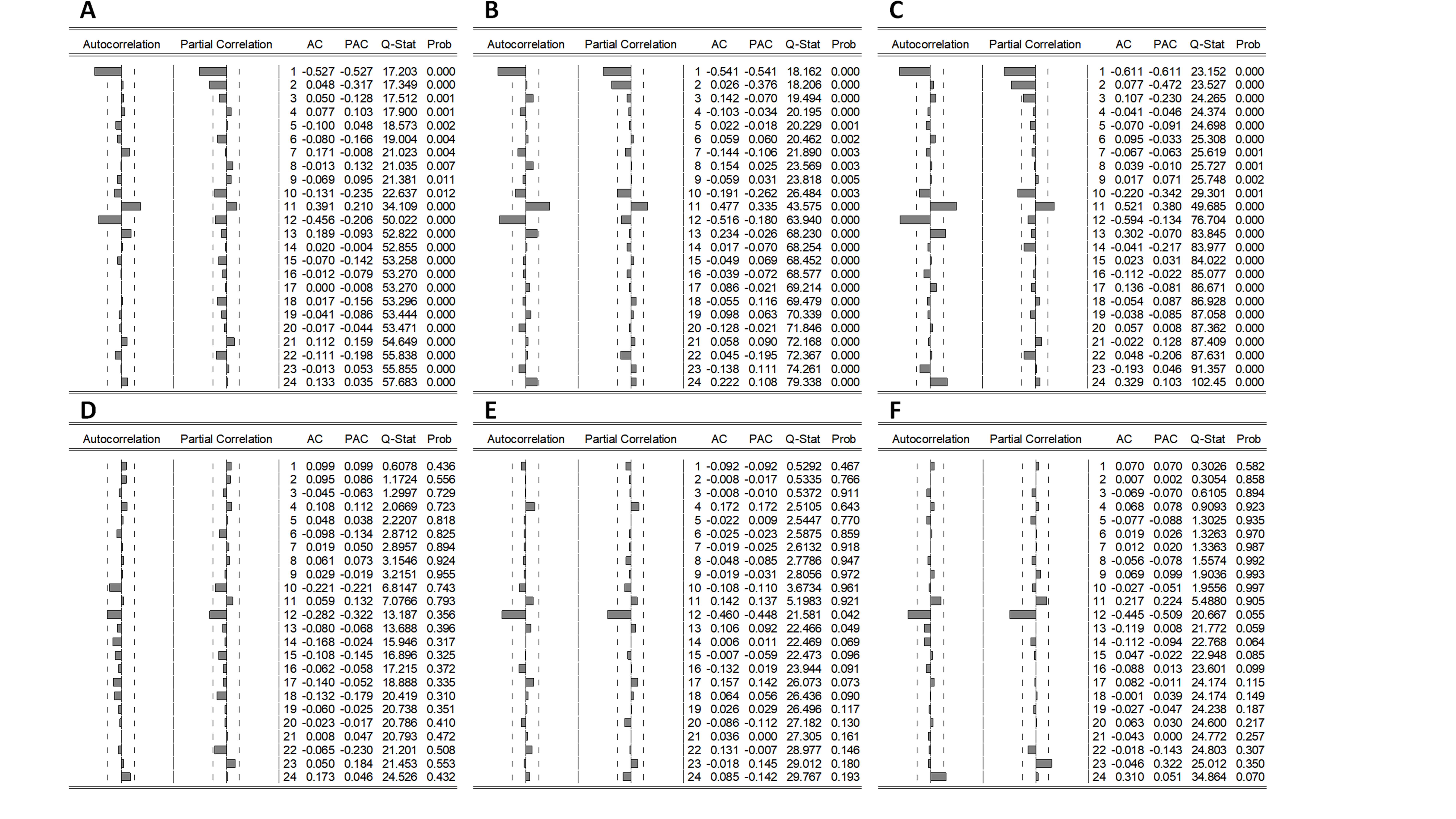

Supplement: Supplementary Figure 4 — ACF and PACF graphs of the post differential and residual sequences from 2011 to 2016. (A) ACF and PACF graphs of the AIDS data (d = 1 and D = 1). (B) ACF and PACF graphs of the gonorrhea data (d = 1 and D = 1). (C) ACF and PACF graphs of the syphilis data (d = 1 and D = 1). (D) ACF and PACF graphs of the residential sequence from AIDS data. (E) ACF and PACF graphs of the residential sequence from gonorrhea data. (F) ACF and PACF graphs of the residential sequence from syphilis data. [file Image_4.JPEG]

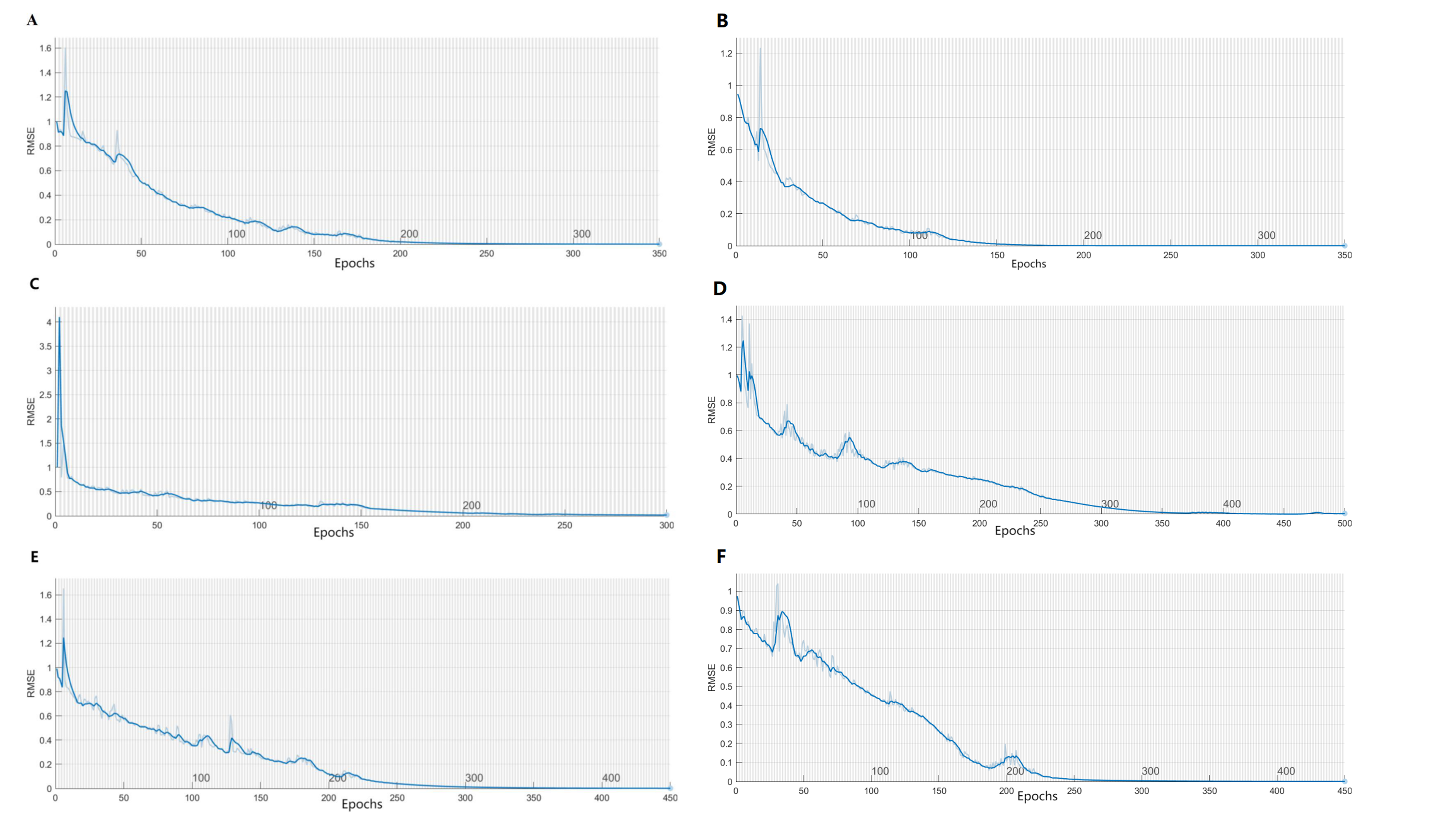

Supplement: Supplementary Figure 5 — Training charts of LSTM model. (A) One-year prediction for AIDS. (B) Five-year prediction for AIDS. (C) One-year prediction for gonorrhea. (D) Five-year prediction for gonorrhea. (E) One-year prediction for syphilis. (F) Five-year prediction for syphilis. [file Image_5.JPEG]
