# Supplementary material for: Development and comparison of predictive models for sexually transmitted diseases—AIDS, gonorrhea, and syphilis in China, 2011–2021
Source: Front Public Health. 2022 Aug 12;10:966813. doi: 10.3389/fpubh.2022.966813 (PMC9450018; doi:10.3389/fpubh.2022.966813)
Supplement: Supplementary file 6 [file Table_1.docx]

| Table S1. Determination of hidden layer neuron number | | | | | |  |  |  |  |  |  |
| --- | --- | --- | --- | --- | --- | --- | --- | --- | --- | --- | --- |
| **STDs** | **ERNN^a^** | |  | **ARIMA-ERNN^a^** | |  | **ERNN^b^** | |  | **ARIMA-ERNN^b^** | |
|  | **Neurons** | **MSE** |  | **Neurons** | **MSE** |  | **Neurons** | **MSE** |  | **Neurons** | **MSE** |
| AIDS | 4 | 0.0386 |  | 2 | 0.0408 |  | 4 | 0.0502 |  | 2 | 0.0496 |
|  | 5 | 0.0398 |  | 3 | 0.0407 |  | 5 | 0.0496 |  | 3 | 0.0474 |
|  | 6 | 0.0388 |  | 4 | 0.0413 |  | 6 | 0.0579 |  | **4** | **0.0473** |
|  | 7 | 0.0387 |  | **5** | **0.0406** |  | 7 | 0.0555 |  | 5 | 0.2212 |
|  | 8 | 0.0429 |  | 6 | 0.0407 |  | 8 | 0.1169 |  | 6 | 0.0485 |
|  | **9** | **0.0381** |  | 7 | 0.0408 |  | 9 | 0.0508 |  | 7 | 0.0521 |
|  | 10 | 0.0393 |  | 8 | 0.0408 |  | 10 | 0.0566 |  | 8 | 0.0478 |
|  | 11 | 0.0390 |  | 9 | 0.0417 |  | 11 | 0.0558 |  | 9 | 0.0491 |
|  | 12 | 0.0391 |  | 10 | 0.0409 |  | 12 | 0.0724 |  | 10 | 0.0475 |
|  | 13 | 0.0392 |  | 11 | 0.0409 |  | **13** | **0.0487** |  | 11 | 0.0491 |
|  |  |  |  |  |  |  |  |  |  |  |  |
| Gonorrhea | 4 | 0.0374 |  | 2 | 0.0238 |  | **4** | **0.0610** |  | 2 | 0.0437 |
|  | 5 | 0.0386 |  | 3 | 0.0237 |  | 5 | 0.0643 |  | 3 | 0.0434 |
|  | 6 | 0.0370 |  | **4** | **0.0234** |  | 6 | 0.0675 |  | 4 | 0.0440 |
|  | 7 | 0.0364 |  | 5 | 0.0246 |  | 7 | 0.0770 |  | 5 | 0.0452 |
|  | 8 | 0.0360 |  | 6 | 0.0237 |  | 8 | 0.0703 |  | 6 | 0.0433 |
|  | 9 | 0.0360 |  | 7 | 0.0251 |  | 9 | 0.0836 |  | **7** | **0.0431** |
|  | 10 | 0.0390 |  | 8 | 0.0234 |  | 10 | 0.0662 |  | 8 | 0.0441 |
|  | 11 | 0.0355 |  | 9 | 0.0238 |  | 11 | 0.0652 |  | 9 | 0.0465 |
|  | **12** | **0.0351** |  | 10 | 0.0235 |  | 12 | 0.0820 |  | 10 | 0.0446 |
|  | 13 | 0.0366 |  | 11 | 0.0241 |  | 13 | 0.0655 |  | 11 | 0.0441 |
|  |  |  |  |  |  |  |  |  |  |  |  |
| Syphilis | 4 | 0.0442 |  | 2 | 0.0398 |  | 4 | 0.0851 |  | 2 | 0.0694 |
|  | 5 | 0.0437 |  | 3 | 0.0441 |  | 5 | 0.1096 |  | 3 | 0.0619 |
|  | 6 | 0.0459 |  | 4 | 0.0402 |  | 6 | 0.0801 |  | 4 | 0.0679 |
|  | **7** | **0.0429** |  | 5 | 0.0400 |  | 7 | 0.0772 |  | 5 | 0.0646 |
|  | 8 | 0.0461 |  | 6 | 0.0400 |  | 8 | 0.0757 |  | 6 | 0.0621 |
|  | 9 | 0.0495 |  | 7 | 0.0455 |  | 9 | 0.1302 |  | 7 | 0.0648 |
|  | 10 | 0.0473 |  | **8** | **0.0397** |  | **10** | **0.0718** |  | 8 | 0.1051 |
|  | 11 | 0.0446 |  | 9 | 0.0400 |  | 11 | 0.0792 |  | **9** | **0.0618** |
|  | 12 | 0.0496 |  | 10 | 0.0400 |  | 12 | 0.0756 |  | 10 | 0.0628 |
|  | 13 | 0.0838 |  | 11 | 0.0399 |  | 13 | 0.0764 |  | 11 | 0.0676 |
| ^a^: one-year prediction. ^b^: five-year prediction. Bold: the optimal number of neurons and MSE. | | | | | | | | | | | |
