# Supplementary material for: Development and comparison of predictive models for sexually transmitted diseases—AIDS, gonorrhea, and syphilis in China, 2011–2021
Source: Front Public Health. 2022 Aug 12;10:966813. doi: 10.3389/fpubh.2022.966813 (PMC9450018; doi:10.3389/fpubh.2022.966813)
Supplement: Supplementary file 7 [file Table_2.docx]

| Table S2. Incidence values of the STDs for 2021 predicted by different forecasting models. | | | | | | | | | | | | | |
| --- | --- | --- | --- | --- | --- | --- | --- | --- | --- | --- | --- | --- | --- |
| **STDs** | **Month** | **Observed** | **ARIMA** | |  | **ERNN** | |  | **ARIMA-ERNN** | |  | **LSTM** | |
|  |  |  | **Predicted** | **RE(%)** |  | **Predicted** | **RE(%)** |  | **Predicted** | **RE(%)** |  | **Predicted** | **RE(%)** |
| AIDS | January | 0.231 | 0.157 | 32.028 |  | 0.210 | 9.219 |  | 0.191 | 17.461 |  | 0.264 | 14.396 |
|  | February | 0.216 | 0.127 | 41.168 |  | 0.162 | 25.214 |  | 0.175 | 19.013 |  | 0.159 | 26.475 |
|  | March | 0.421 | 0.303 | 27.970 |  | 0.341 | 19.174 |  | 0.302 | 28.340 |  | 0.379 | 10.030 |
|  | April | 0.374 | 0.357 | 4.549 |  | 0.385 | 3.007 |  | 0.344 | 8.036 |  | 0.358 | 4.273 |
|  | May | 0.357 | 0.317 | 11.222 |  | 0.384 | 7.542 |  | 0.322 | 10.025 |  | 0.426 | 19.267 |
|  | June | 0.423 | 0.417 | 1.572 |  | 0.482 | 13.873 |  | 0.395 | 6.618 |  | 0.453 | 7.013 |
|  | July | 0.239 | 0.373 | 56.143 |  | 0.402 | 68.299 |  | 0.370 | 54.958 |  | 0.395 | 65.566 |
|  | August | 0.333 | 0.303 | 9.107 |  | 0.416 | 24.839 |  | 0.327 | 1.985 |  | 0.456 | 36.661 |
|  | September | 0.357 | 0.405 | 13.419 |  | 0.492 | 37.789 |  | 0.402 | 12.703 |  | 0.424 | 18.755 |
|  | October | 0.379 | 0.272 | 28.386 |  | 0.361 | 4.740 |  | 0.315 | 16.906 |  | 0.366 | 3.595 |
|  | November | 0.460 | 0.345 | 24.855 |  | 0.400 | 13.055 |  | 0.371 | 19.245 |  | 0.500 | 8.727 |
|  | December | 0.530 | 0.378 | 28.641 |  | 0.445 | 16.118 |  | 0.399 | 24.747 |  | 0.484 | 8.805 |
|  |  |  |  |  |  |  |  |  |  |  |  |  |  |
| Gonorrhea | January | 0.728 | 0.603 | 17.241 |  | 0.667 | 8.371 |  | 0.593 | 18.546 |  | 0.749 | 2.815 |
|  | February | 0.542 | 0.259 | 52.101 |  | 0.480 | 11.390 |  | 0.311 | 42.544 |  | 0.580 | 7.147 |
|  | March | 0.770 | 0.339 | 55.987 |  | 0.411 | 46.608 |  | 0.381 | 50.557 |  | 0.651 | 15.522 |
|  | April | 0.770 | 0.456 | 40.737 |  | 0.426 | 44.653 |  | 0.482 | 37.420 |  | 0.775 | 0.723 |
|  | May | 0.763 | 0.591 | 22.532 |  | 0.496 | 34.987 |  | 0.597 | 21.703 |  | 0.799 | 4.783 |
|  | June | 0.775 | 0.678 | 12.496 |  | 0.571 | 26.324 |  | 0.673 | 13.136 |  | 0.858 | 10.616 |
|  | July | 0.832 | 0.777 | 6.637 |  | 0.655 | 21.200 |  | 0.758 | 8.804 |  | 0.863 | 3.776 |
|  | August | 0.851 | 0.785 | 7.740 |  | 0.760 | 10.687 |  | 0.769 | 9.622 |  | 0.861 | 1.125 |
|  | September | 0.832 | 0.854 | 2.653 |  | 0.778 | 6.407 |  | 0.829 | 0.244 |  | 0.871 | 4.775 |
|  | October | 0.759 | 0.775 | 2.050 |  | 0.761 | 0.202 |  | 0.767 | 1.086 |  | 0.804 | 5.871 |
|  | November | 0.787 | 0.828 | 5.143 |  | 0.805 | 2.245 |  | 0.815 | 3.516 |  | 0.763 | 3.109 |
|  | December | 0.798 | 0.861 | 7.909 |  | 0.823 | 3.252 |  | 0.846 | 6.043 |  | 0.804 | 0.869 |
|  |  |  |  |  |  |  |  |  |  |  |  |  |  |
| Syphilis | January | 2.838 | 2.595 | 8.550 |  | 2.788 | 1.744 |  | 2.658 | 6.348 |  | 2.973 | 4.762 |
|  | February | 2.380 | 1.431 | 39.885 |  | 2.024 | 14.962 |  | 1.610 | 32.374 |  | 2.795 | 17.436 |
|  | March | 3.588 | 2.829 | 21.155 |  | 2.749 | 23.399 |  | 2.861 | 20.280 |  | 3.664 | 2.117 |
|  | April | 3.477 | 3.321 | 4.485 |  | 2.997 | 13.815 |  | 3.299 | 5.138 |  | 3.533 | 1.587 |
|  | May | 3.398 | 3.287 | 3.281 |  | 3.200 | 5.835 |  | 3.264 | 3.948 |  | 3.554 | 4.585 |
|  | June | 3.358 | 3.338 | 0.588 |  | 3.247 | 3.304 |  | 3.307 | 1.524 |  | 3.536 | 5.300 |
|  | July | 3.649 | 3.578 | 1.957 |  | 3.447 | 5.539 |  | 3.518 | 3.589 |  | 3.739 | 2.478 |
|  | August | 3.263 | 3.371 | 3.303 |  | 3.378 | 3.497 |  | 3.329 | 2.021 |  | 3.736 | 14.493 |
|  | September | 3.242 | 3.490 | 7.638 |  | 3.566 | 9.992 |  | 3.432 | 5.863 |  | 3.099 | 4.422 |
|  | October | 2.896 | 3.206 | 10.714 |  | 3.279 | 13.235 |  | 3.174 | 9.614 |  | 3.419 | 18.078 |
|  | November | 2.986 | 3.238 | 8.427 |  | 3.342 | 11.918 |  | 3.199 | 7.134 |  | 2.781 | 6.874 |
|  | December | 2.996 | 3.224 | 7.609 |  | 3.218 | 7.401 |  | 3.184 | 6.249 |  | 3.039 | 1.416 |
